# Adsorption Characteristics and Mechanistic Role of Ionic Species on the Chalcopyrite (112) Surface Based on DFT Simulations

**DOI:** 10.3390/ijms27094012

**Published:** 2026-04-30

**Authors:** Luis Rios-Colque, Pedro A. Robles, Gonzalo R. Quezada, Victor Rios-Colque

**Affiliations:** 1 Doctorado en Industria Inteligente, Facultad de Ingeniería, Pontificia Universidad Católica de Valparaíso, Valparaíso 2340000, Chile; luis.rios.c@mail.pucv.cl (L.R.-C.); victor.rios.c@mail.pucv.cl (V.R.-C.); 2 Escuela de Ingeniería Química, Pontificia Universidad Católica de Valparaíso, Valparaíso 2340000, Chile; 3 Escuela de Ingeniería Química, Universidad del Bio-Bio, Concepción 4030000, Chile; grquezada@ubiobio.cl

**Keywords:** chalcopyrite (112) surface, ionic species adsorption, adsorption characteristics, low-quality water, density functional theory

## Abstract

The increasing scarcity of freshwater in mining regions of Chile has promoted the use of low-quality water as an alternative in flotation processes, significantly modifying their operating conditions. In particular, high salt concentrations and the presence of dissolved ionic species may interfere with the adsorption of collectors on chalcopyrite, thereby reducing its hydrophobicity. In this context, the present study analyzes the adsorption characteristics and the mechanistic role of selected representative ionic species on the chalcopyrite surface. To this end, simulations based on density functional theory (DFT) were employed to describe the interaction between the chalcopyrite (112) surface and Na^+^, Ca^2+^, Mg^2+^, and OH^−^ ions. After geometric convergence of the optimized structures was achieved, adsorption energies, charge redistribution based on Mulliken population analysis, and the final structural configurations were evaluated for each case. The results revealed clearly differentiated behaviors among the ionic species considered. The OH^−^ ion exhibited a localized and specific interaction with metal-centered sites. By contrast, Ca^2+^ and Mg^2+^ show stable adsorption near sulfur atoms, indicating a higher affinity that may lead to the occupation or blocking of active surface sites. Meanwhile, Na^+^ displays a weak interaction without inducing significant structural modifications. Overall, these findings provide an atomistic-level interpretation of how ionic species present in low-quality water can influence the surface reactivity of chalcopyrite under flotation operating conditions.

## 1. Introduction

Copper mining constitutes a central activity for the Chilean economy and the supply of this metal, whose demand has increased in recent decades due to its use in technologies associated with the energy transition [1,2,3]. Primary production relies on the exploitation of mineral deposits, predominantly sulfide ores, whose processing is constrained by declining ore grades and by operational restrictions derived from environmental requirements, particularly in terms of water and energy use [1,2].

Among sulfide ores, chalcopyrite (CuFeS_2_) is the most important, despite having a lower theoretical copper content (34.6%) than other minerals such as bornite (Cu_5_FeS_4_), chalcocite (Cu_2_S), or covellite (CuS) [1,4]. Its predominance in industry is explained by its wide geological distribution, the occurrence of large deposits that enable large-scale exploitation, its compatibility with established metallurgical routes, and its frequent association with valuable by-products [5,6].

Chalcopyrite is commonly associated with non-sulfide gangue minerals, such as silicates and clays, as well as with other accompanying sulfides, including pyrite (FeS_2_) [4,7]. This association makes its concentration by froth flotation necessary, a physicochemical process of selective separation that takes place in an aqueous medium, in which chalcopyrite particles attach to air bubbles and float, whereas the gangue remains in the pulp and is discarded [8,9,10].

In practice, flotation requires the addition of reagents to control the process. Among these, collectors play a key role, as they adsorb onto specific sites on the chalcopyrite surface, rendering it more hydrophobic and thereby promoting its recovery as a valuable mineral [10,11]. In a complementary manner, other reagents, including pH regulators, depressants, and activators, are employed to adjust operating conditions, optimize separation performance, and improve concentrate quality [10,12].

The problem under consideration lies in the growing scarcity of freshwater in mining regions of Chile, which has driven the progressive use of seawater as an alternative resource in flotation processes [13,14]. However, its low quality significantly reduces process efficiency, forcing mining operations to treat larger volumes of material, thereby increasing energy consumption, waste generation, operating costs, and the associated environmental impact [13,15].

To quantify the associated economic impact, copper mining represented approximately 10% of the Chilean Gross Domestic Product (GDP) and exceeded 50% of total national exports by the conclusion of 2024 [16]. Given that annual production reached nearly 5.5 million tons during the same period [17], even marginal increments in flotation efficiency may generate substantial increases in aggregate output and sectoral profitability. Against this backdrop, the identified problem transcends specific operational instances and acquires a structural dimension for the Chilean mining industry.

Numerous experimental studies have demonstrated that the elevated concentration of dissolved ionic species in seawater—primarily composed of Na^+^ (~0.486 mol kg^−1^), Cl^−^ (~0.566 mol kg^−1^), Mg^2+^ (~0.055 mol kg^−1^), SO_4_^2−^ (~0.029 mol kg^−1^), and Ca^2+^ (0.011 mol kg^−1^)—may affect chalcopyrite recovery, even under controlled conditions [18,19,20]. On one hand, monovalent cations such as Na^+^ have been reported to facilitate flotation under neutral to moderately alkaline pH conditions (~7–9) and at moderate ionic concentrations, where the formation of secondary species is limited [21,22].

As reported by several authors, this phenomenon is ascribed to the compression of the electrical double layer, which diminishes the electrostatic repulsion between particles and bubbles, thereby facilitating their adhesion [13,21,23]. This, in turn, leads to an enhancement of the apparent hydrophobicity of chalcopyrite, suggesting a predominantly physico-electrostatic mechanism [21,24]. However, this behavior is attenuated at high ionic strengths or when the formation of hydrolyzed species begins to govern the interfacial interactions [25,26].

On the other hand, divalent cations such as Ca^2+^ and Mg^2+^ may adversely affect chalcopyrite flotation, particularly under alkaline conditions. In this regime, their hydrolysis promotes the formation of hydroxylated species and precipitates that adsorb onto the mineral surface, thereby increasing its hydrophilicity [22,26,27]. This effect is more pronounced for Mg^2+^, as its derived species exhibit a greater propensity to form stable surface coatings [21,28,29].

Nevertheless, under moderate pH conditions and in the absence of significant precipitation, Ca^2+^ has been reported to exhibit neutral or even slightly favorable behavior, linked to modifications in surface charge and the attenuation of electrostatic repulsion [21,27]. From this standpoint, the role of metal ions cannot be fully captured by a single mechanism; instead, it reflects the combined influence of ionic speciation, the formation of hydroxylated species, and interfacial interactions, all ultimately controlled by pH, ionic strength, and system composition [13,22].

At present, computational simulations based on density functional theory (DFT) offer a complementary approach for investigating the specific interactions between ionic species and well-defined mineral surfaces. These methods enable direct atomistic-scale examination of adsorption energies, electronic charge redistribution, and stable structural configurations, among other relevant descriptors [11,30].

In this vein, recent studies have shifted toward complementing the aforementioned experimental evidence and elucidating the underlying interaction mechanisms at the atomic scale using DFT. For instance, Luo et al. examined the influence of CaOH^+^, reporting favorable adsorption on the chalcopyrite surface associated with the formation of Ca–S bonds; additional electronic structure analyses were conducted to demonstrate the nature of the interaction at the orbital level [31].

Along similar lines, Li et al. used adsorption energies to support the notion that an interaction between Mg(OH)_2_ and chalcopyrite is sufficient to promote the formation of hydrophilic surface coatings [32]. In general, these studies focus on pre-hydrolyzed or precipitated species; consequently, they provide limited insight into the fundamental ion–surface interactions that precede such processes.

Despite these advances, limitations remain in understanding the mechanisms governing metal ion behavior in chalcopyrite flotation due to the coexistence of multiple species in equilibrium and the overlap of their effects. Therefore, it is necessary to investigate ions individually to disentangle ion–surface interactions and establish a mechanistic basis for interpreting subsequent speciation processes.

On this basis, the present study aims to analyze the interaction of selected ionic species—Na^+^, Ca^2+^, Mg^2+^, and OH^−^—with the chalcopyrite surface through DFT simulations in order to identify differentiated adsorption patterns and their implications for surface reactivity. The chalcopyrite (112) surface was selected due to its relative stability and its exposure of coordinatively unsaturated sites, which favor the adsorption of these species and make it a representative plane for the study of interfacial processes [33,34].

It is worth noting that, although anions such as Cl^−^ and SO_4_^2−^ are also present in seawater, their role is primarily associated with ionic strength, ion pairing, and solution chemistry, rather than with specific interactions with the mineral surface [13,35]. Thus, their exclusion is intended to isolate the effect of metal cations, avoiding overlapping contributions that may hinder the identification of intrinsic adsorption mechanisms.

The obtained energetic, electronic, and structural findings are expected to provide mechanistic insights that support a fundamental interpretation of the ionic effects observed experimentally in flotation systems operating with low-quality water, such as those encountered in copper mining in Chile. The remainder of this paper is organized as follows: Section 2 describes the computational methodology, Section 3 presents the results, Section 4 discusses the findings, and Section 5 outlines the main conclusions.

## 2. Materials and Methods

The calculations based on density functional theory (DFT) were performed using the CASTEP (Cambridge Serial Total Energy Package) program version 19.11, Cambridge, UK [36]. The exchange–correlation interactions were described within the Generalized Gradient Approximation (GGA) using the Perdew–Burke–Ernzerhof (PBE) functional. Electron–nucleus interactions were represented using ultrasoft pseudopotentials. The electronic wave functions were expanded in a plane-wave basis set with a cutoff energy of 351 eV. Brillouin zone sampling was performed using the Monkhorst–Pack scheme with a 2 × 2 × 1 k-point mesh.

Geometry optimization was conducted using the Broyden–Fletcher–Goldfarb–Shanno (BFGS) minimization algorithm with line search. The convergence criteria for geometry optimization were defined as 2 × 10^−5^ eV/atom for energy, 0.05 eV/Å for maximum force, 0.002 Å for maximum displacement, and 0.1 GPa for maximum stress. Self-consistent field (SCF) convergence tolerance was defined as 1 × 10^−6^ eV/atom.

Spin-polarized calculations were performed to account for the magnetic nature of chalcopyrite. Initial magnetic moments of 4 μB were assigned to Fe atoms, consistent with a high-spin Fe^3+^ configuration, and were optimized within the SCF procedure.

The structure used in this study was based on the crystal structure of chalcopyrite (CuFeS_2_) reported in [33]. Chalcopyrite crystallizes in a tetragonal system with space group I-42d and lattice parameters a = b = 5.289 Å and c = 10.423 Å. Based on this structure, the (112) surface was constructed for the adsorption simulations. This surface was selected as a commonly reported cleavage plane of chalcopyrite, exhibiting relative stability and exposing coordinatively unsaturated atoms suitable for studying adsorption and ion–surface interactions [33,34].

The surface slab was generated using a 2 × 2 × 1 supercell, introducing a 10 Å vacuum layer perpendicular to the surface to avoid interactions between periodic images. No explicit convergence tests were performed for the slab thickness; however, the model employed is consistent with configurations commonly reported in the literature for sulfide ores, which are generally considered sufficient to represent bulk-like behavior in the inner layers [37,38,39].

The surface structure was optimized through geometric relaxation of the supercell prior to the adsorption calculations. The ionic species considered were Na^+^, Ca^2+^, Mg^2+^, and OH^−^, modeled with their respective formal charges. The initial adsorption configurations were generated by positioning the ionic species near surface metal sites while maintaining physically reasonable initial distances to avoid unrealistic atomic overlaps.

The relative stability of the adsorbed configurations was evaluated through the calculation of adsorption energies, defined as follows:

(1) $$E_{ads}=E_{adsorbate/surface}-(E_{adsorbate}+E_{surface})$$

where $E_{ads}$ is the adsorption energy, $E_{adsorbate/surface}$ is the total energy of the adsorbate-surface system, $E_{surface}$ is the energy of the clean surface, and $E_{adsorbate}\;$ is the energy of the isolated adsorbate. More negative values of $E_{ads}$ indicate stronger interactions between the ionic species and the mineral surface.

Subsequently, a Mulliken population analysis was performed to evaluate the charge redistribution induced by adsorption and to understand the nature of the ion–surface interactions at the atomic scale. Also, partial density of states (PDOS) calculations were carried out for the ion with the strongest adsorption in order to analyze the electronic structure and the orbital interactions governing the adsorption mechanism.

## 3. Results

### 3.1. Optimized Structure of the Chalcopyrite (112) Surface

**Figure 1:**
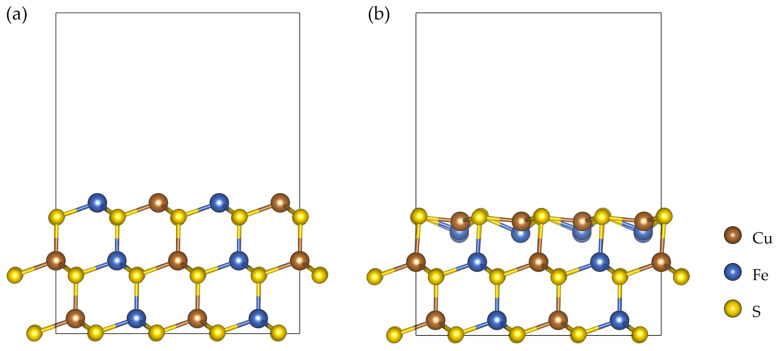
CuFeS_2_ (112) slab model: (**a**) initial structure and (**b**) optimized structure.

Figure 1 shows the initial structure of the CuFeS_2_ (112) slab and the optimized structure obtained after geometry optimization.

After optimization, the atoms in the surface layer undergo a slight displacement relative to their initial positions. Nevertheless, the overall slab structure remains stable and preserves the characteristic connectivity of the Cu–S and Fe–S tetrahedra typical of chalcopyrite. In this way, the suitability of the surface model used for the subsequent adsorption calculations of ionic species is confirmed.

### 3.2. Adsorption of Ionic Species on the Chalcopyrite (112) Surface

**Figure 2:**
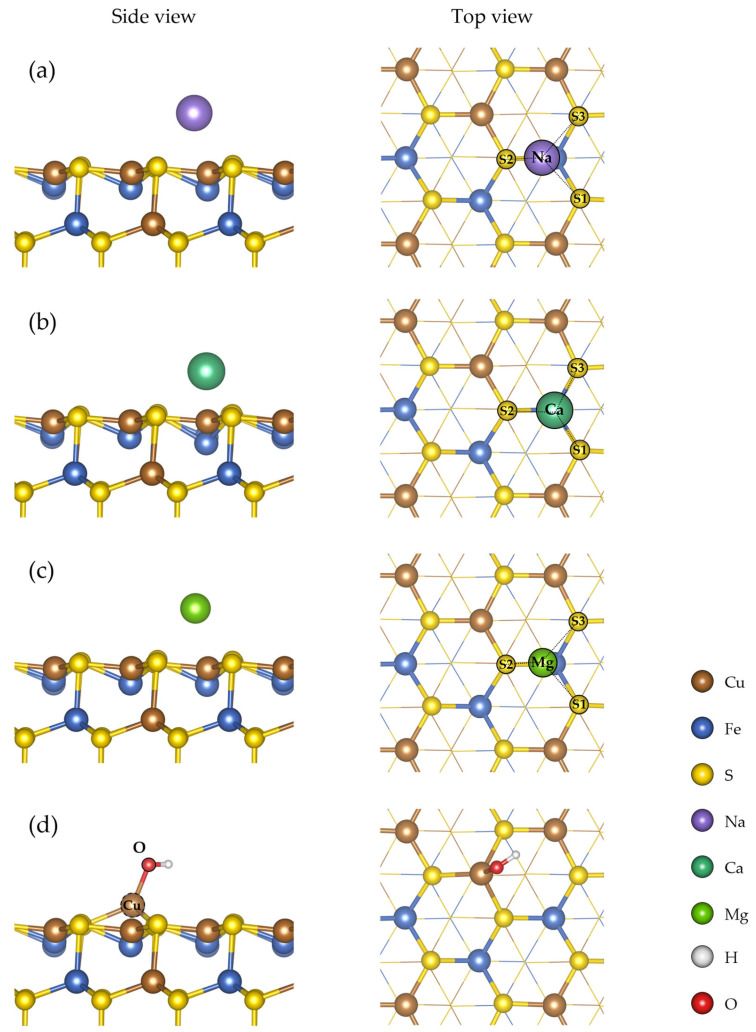
Optimized adsorption configurations of ionic species on the CuFeS_2_ (112) surface: (**a**) Na^+^, (**b**) Ca^2+^, (**c**) Mg^2+^, and (**d**) OH^−^.

The optimized adsorption configurations of the ionic species Na^+^, Ca^2+^, Mg^2+^, and OH^−^ on the CuFeS_2_ (112) surface are shown in Figure 2.

After geometry optimization, the ionic species adopt different positions relative to the chalcopyrite surface. The Na^+^ remains at a relatively larger distance from the surface compared to the other species, indicating a weak interaction with surface atoms. In contrast, the Ca^2+^ and Mg^2+^ cations are preferentially located closer to sulfur atoms in the surface layer, suggesting stronger adsorption. In the case of OH^−^, the oxygen atom is oriented toward surface metal atoms after structural relaxation, indicating a specific interaction with metal-centered sites.

The adsorption energies calculated for each ionic species on the CuFeS_2_ surface are presented in Table 1, providing a quantitative comparison of their interaction strength with the mineral surface.

As can be observed, all adsorption energies are negative, indicating that the adsorption of the ionic species on the CuFeS_2_ surface is thermodynamically favorable. A clear difference in adsorption strength is observed among the species. In particular, Ca^2+^ and Mg^2+^ exhibit significantly more negative adsorption energies than Na^+^ and OH^−^, reflecting a stronger affinity for the surface. Among the species considered, Na^+^ shows the weakest interaction, whereas OH^−^ displays an intermediate adsorption strength.

### 3.3. Mulliken Population Analysis

Mulliken population analysis was performed to evaluate the electronic redistribution associated with the adsorption of ionic species on the CuFeS_2_ (112) surface. The Mulliken charges of the relevant atoms before and after adsorption are presented in Table 2.

For Na^+^, the variations in the charges of both the adsorbed ion and the neighboring surface atoms are relatively small, indicating limited electronic redistribution. In contrast, the Ca^2+^ and Mg^2+^ cations exhibit more pronounced changes in their charges after adsorption, accompanied by noticeable variations in the charges of the adjacent sulfur atoms. In turn, the adsorption of OH− is associated with changes in the charge of the oxygen atom and the nearest surface metal atom after surface relaxation.

Additionally, the Mulliken bond populations and the interatomic distances between the adsorbed species and the nearest surface atoms are presented in Table 3, providing further insight into the nature and strength of the adsorption interactions.

For Na^+^, the interaction with surface sulfur atoms is characterized by relatively long interatomic distances and low Mulliken population values. For Ca^2+^, the interactions with sulfur atoms show shorter distances and higher population values compared to Na^+^. In the case of Mg^2+^, the distances to sulfur atoms are comparable to those of Ca^2+^; however, the corresponding Mulliken population values are lower. For OH^−^, the interaction between the oxygen atom and a surface Cu atom exhibits a shorter interatomic distance than those observed for the cationic species, together with a higher Mulliken population value.

### 3.4. Projected Density of States (PDOS) Analysis

**Figure 3:**
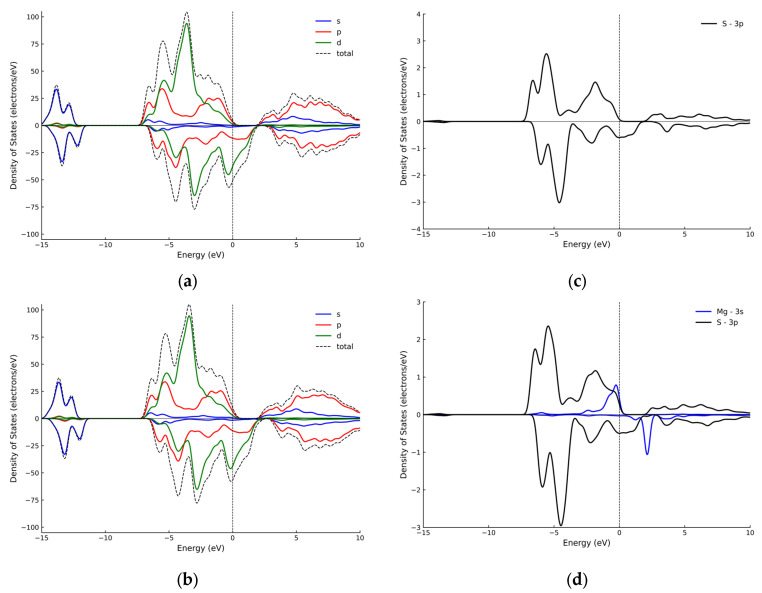
Total and projected density of states (DOS and PDOS) of the CuFeS_2_ (112) surface before and after Mg^2+^ adsorption: (**a**) total DOS before adsorption; (**b**) total DOS after adsorption; (**c**) S 3p PDOS before adsorption; (**d**) Mg-3s and S-3p PDOS after adsorption.

To further elucidate the nature of the ion–surface interactions, projected density of states (PDOS) calculations were performed for the most strongly adsorbed ion. This analysis provides insight into the electronic structure and the orbital hybridization between the adsorbate and the surface atoms. The corresponding total and projected density of states before and after Mg^2+^ adsorption are presented in Figure 3.

The comparison between the total DOS of the surface before and after adsorption (Figure 3a,b) shows only minor differences in the overall electronic structure, suggesting that the presence of Mg^2+^ does not substantially affect the global electronic properties of the system. In particular, no significant changes are observed in the distribution of states near the Fermi level.

In contrast, the PDOS analysis (Figure 3c,d) provides insight into local electronic effects. The S 3p orbitals dominate the valence region in both cases, while after adsorption, the contribution of Mg 3s states remains relatively small and exhibits limited overlap with the S 3p orbitals. These observations indicate that the interaction is mainly localized, with no clear evidence of strong orbital hybridization.

Together with the Mulliken charge analysis and the low bond population values, these results suggest that the interaction between Mg^2+^ and the surface is predominantly electrostatic in nature.

## 4. Discussion

The adsorption behavior of ionic species on the CuFeS_2_ (112) surface can be interpreted based on the optimized geometric configurations, adsorption energies, and charge variations obtained from Mulliken population analysis. Unlike previous studies that focus on hydrolyzed or precipitated species, the present results isolate intrinsic ion–surface interactions prior to speciation, providing an atomistic description of the initial stages of adsorption.

In the case of Na^+^, the larger distance from surface sulfur atoms and the low Mulliken population values indicate a weak interaction with limited electronic redistribution. Although the adsorption energy is negative, its magnitude should be interpreted comparatively, as DFT calculations involving isolated charged species may overestimate absolute values. Therefore, structural and electronic descriptors provide a more reliable basis for assessing interaction strength. This behavior is consistent with predominantly electrostatic adsorption, suggesting that Na^+^ does not directly modify active sites but rather influences interfacial properties such as double-layer compression [21,23,24,25,26].

In contrast, Ca^2+^ and Mg^2+^ exhibit shorter distances to surface sulfur atoms and more pronounced charge redistribution in nearby atoms. These features indicate a stronger interaction with the chalcopyrite surface. The significantly more negative adsorption energies obtained for these cations further support their higher affinity for the surface. Although the Mulliken population values for Mg^2+^ remain relatively low, the substantial charge transfer and adsorption energy indicate that the interaction is predominantly ionic in nature, with a limited covalent contribution. From a mechanistic perspective, these results suggest that divalent cations may occupy or partially block adsorption sites even in the absence of hydrolysis, providing an atomistic explanation for their experimentally observed depressing effects under saline conditions [22,26,27,28,29].

The adsorption of OH^−^ shows a behavior distinct from that of the cationic species. The optimized configuration indicates that the oxygen atom is oriented toward a surface metal atom with a short interatomic distance and relatively high Mulliken population value. These features suggest a localized interaction involving direct coordination with metal sites, rather than purely electrostatic stabilization. This behavior is consistent with the initial stages of surface hydroxylation processes reported under alkaline conditions [29,31].

Taken together, these observations indicate that the adsorption mode depends not only on ionic charge but also on the specific interaction with surface sites. Divalent cations such as Ca^2+^ and Mg^2+^ preferentially stabilize near sulfur atoms, whereas Na^+^ exhibits a weaker interaction dominated by electrostatic effects. In contrast, OH^−^ preferentially interacts with surface metal centers, indicating a distinct adsorption mechanism. These results highlight that adsorption strength cannot be interpreted solely in terms of adsorption energy but must also consider electronic structure and bonding characteristics.

To complement this analysis, the PDOS results for Mg^2+^ provide insight into local electronic effects. The S-3p orbitals dominate the valence region, while the contribution of Mg-3s states is relatively small and shows limited overlap with S-3p orbitals. This indicates that the interaction is largely localized and lacks significant orbital hybridization. Consistent with the Mulliken analysis, this supports a predominantly ionic interaction. Similar behavior is expected for Ca^2+^ and Na^+^, where adsorption is mainly governed by electrostatic effects.

From an applied perspective, these findings provide a mechanistic basis for interpreting the impact of ionic species on chalcopyrite flotation under saline conditions. The strong affinity of Ca^2+^ and Mg^2+^ for surface sites suggests that these ions may reduce collector efficiency by limiting the availability of reactive sites, even prior to hydrolysis or precipitation. This effect is particularly relevant in Chilean copper mining, where the increasing use of seawater introduces high concentrations of divalent cations into flotation circuits.

Following this reasoning, Na^+^ is expected to influence flotation primarily through modifications of the physicochemical properties of the pulp, such as ionic strength and double-layer compression, rather than through direct competition for surface sites. Additionally, hydroxyl species may promote progressive surface hydroxylation, which can modify surface reactivity and affect collector adsorption under alkaline conditions.

Overall, these results demonstrate that ionic species present in saline solutions influence the interfacial behavior of chalcopyrite through distinct interaction mechanisms. In particular, divalent cations such as Ca^2+^ and Mg^2+^ can limit the availability of surface sites for collector adsorption, whereas Na^+^ acts mainly through physicochemical effects in the surrounding medium. These findings provide a mechanistic framework for interpreting experimentally observed flotation responses in seawater systems.

Regarding the limitations of this study, the ionic species considered were modeled as individual entities interacting directly with the mineral surface. In real flotation systems, ions are fully hydrated and surrounded by structured solvation shells. Therefore, their adsorption may involve partial or complete desolvation, associated with an energetic penalty that is not explicitly considered. In this sense, it would be interesting to incorporate this effect in future work in order to evaluate its influence on adsorption energies and the relative stability of the adsorbed configurations.

Likewise, the chalcopyrite surface considered corresponds to an ideal, defect-free model. In natural systems, the presence of impurities, trace elements, structural defects, and surface heterogeneities can modify the distribution of active sites, alter local electronic properties, and affect the interaction with ionic species and flotation reagents. Consequently, future studies should incorporate more representative conditions to better approximate the behavior observed in industrial systems.

## 5. Conclusions

The present study provides an atomistic-level characterization of the interaction between representative ionic species present in low-quality waters and the CuFeS_2_ (112) surface using DFT simulations. By isolating intrinsic ion–surface interactions prior to speciation, the results offer a fundamental framework to understand the initial stages of adsorption.

The findings reveal that ionic species interact with the chalcopyrite surface through distinct mechanisms. OH^−^ exhibits a localized and specific interaction with surface metal centers, consistent with the onset of surface hydroxylation. In contrast, Ca^2+^ and Mg^2+^ show stable adsorption near sulfur sites, consistent with their more negative adsorption energies, indicating a higher affinity that may lead to the occupation or blocking of active surface sites. Meanwhile, Na^+^ displays a weak, predominantly electrostatic interaction with limited impact on the surface structure.

These results highlight that adsorption behavior cannot be interpreted solely in terms of adsorption energy but must also consider the nature of the interaction and the associated electronic structure. In this context, divalent cations are likely to directly influence surface reactivity, whereas monovalent ions primarily affect the interfacial properties of the surrounding medium.

From an applied perspective, the identified interaction patterns provide a mechanistic basis to interpret the effects of saline water on chalcopyrite flotation, particularly under conditions relevant to Chilean copper mining. The results suggest that Ca^2+^ and Mg^2+^ may reduce collector efficiency by limiting the availability of active sites, while OH^−^ may promote surface hydroxylation under alkaline conditions, affecting mineral–reagent interactions.

Although the simulations were conducted on an ideal, defect-free surface and without explicit solvation, the trends obtained offer valuable insight into the role of ionic species in flotation systems. Future work should incorporate more realistic interfacial conditions, including hydration effects, surface heterogeneity, and competitive adsorption with flotation reagents, to further bridge atomistic modeling with industrial processes.

## Figures and Tables

**Table 1:** Calculated adsorption energies of ionic species on the CuFeS_2_ (112) surface.

Adsorbate	Main Interaction	Adsorption Energy (eV)
Na^+^	Na–S	−1.69
Ca^2+^	Ca–S	−16.17
Mg^2+^	Mg–S	−15.70
OH^−^	O–Cu	−2.55

**Table 2:** Mulliken charges of atoms before and after adsorption.

Adsorbate	Atom	Charge Before (e)	Charge After (e)	Δ Charge (e)
Na^+^	Na	1.00	0.93	−0.07
	S1	−0.36	−0.40	−0.04
	S2	−0.35	−0.45	−0.10
	S3	−0.34	−0.37	−0.03
Ca^2+^	Ca	2.00	1.34	−0.66
	S1	−0.36	−0.46	−0.10
	S2	−0.35	−0.46	−0.11
	S3	−0.34	−0.43	−0.09
Mg^2+^	Mg	2.00	0.74	−1.26
	S1	−0.36	−0.38	−0.02
	S2	−0.35	−0.47	−0.12
	S3	−0.34	−0.36	−0.02
OH^−^	O	−1.44	−0.80	0.64
	Cu	0.32	0.61	0.29

**Table 3:** Mulliken population and bond length of the optimized adsorption configurations.

Adsorbate	Interaction	Distance (Å)	Population
Na^+^	Na–S1	3.24	−0.01
	Na–S2	2.72	0.04
	Na–S3	3.28	−0.01
Ca^2+^	Ca–S1	2.69	0.18
	Ca–S2	2.70	0.18
	Ca–S3	2.76	0.15
Mg^2+^	Mg–S1	3.24	−0.06
	Mg–S2	2.68	−0.08
	Mg–S3	3.26	−0.07
OH^−^	O–Cu	1.84	0.40

## Data Availability

The original contributions presented in this study are included in the article. Further inquiries can be directed to the corresponding author.

## References

[1] Schlesinger M., Sole K., Davenport W., Alvear G. (2021). Extractive Metallurgy of Copper.

[2] U.S. Geological Survey (2024). Mineral Commodity Summaries 2024.

[3] Abbas S., Saqib N., Shahzad U. (2024). Global export flow of Chilean copper: The role of environmental innovation and renewable energy transition. Geosci. Front..

[4] Castellón C.I., Toro N., Gálvez E., Robles P., Leiva W.H., Jeldres R.I. (2022). Froth Flotation of Chalcopyrite/Pyrite Ore: A Critical Review. Materials.

[5] Crespo J., Reich M., Barra F., Verdugo J.J., Martínez C., Leisen M., Romero R., Morata D., Marquardt C. (2020). Occurrence and Distribution of Silver in the World–Class Río Blanco Porphyry Cu–Mo Deposit, Central Chile. Econ. Geol..

[6] Hao J., Liu J., Yu Y., Gao H., Qin X., Bai X. (2023). Depressants for separation of chalcopyrite and molybdenite: Review and prospects. Miner. Eng..

[7] Qin S., Yang L., Song H., Dou S., Ma S., Hu Y., Zhao H. (2025). Mechanistic insights into particle interactions and gangue mineral migration in chalcopyrite flotation: Towards efficient copper mineral recovery from polymetallic ores. Appl. Surf. Sci..

[8] Jeldres R.I., Uribe L., Cisternas L.A., Gutierrez L., Leiva W.H., Valenzuela J. (2019). The effect of clays minerals on the process of flotation of copper ores: A critical review. Appl. Clay. Sci..

[9] Fuerstenau M.C., Jameson G.J., Yoon R.H. (2007). Froth Flotation: A Century of Innovation.

[10] Bulatovic S.M. (2007). Handbook of Flotation Reagents: Chemistry, Theory and Practice Flotation of Sulfide Ores.

[11] Rios L.A., Barraza M.J., Robles P.A., Quezada G.R. (2025). Chalcopyrite Flotation, Molecular Design and Smart Industry: A Review. Int. J. Mol. Sci..

[12] Saim A.K., Darteh F.K. (2022). Eco-Friendly and Biodegradable Depressants in Chalcopyrite Flotation: A Review. Miner. Process. Extr. Metall. Rev..

[13] Castro S., Laskowski J.S. (2011). Froth flotation in saline water. KONA Powder Part. J..

[14] Cisternas L.A., Gálvez E.D. (2018). The use of seawater in mining. Miner. Process. Extr. Metall. Rev..

[15] Rao F., Lázaro I., Ibarra L.A. (2017). Solution chemistry of sulphide mineral flotation in recycled water and sea water: A review. Miner. Process. Extr. Metall..

[16] Comisión Chilena del Cobre (Cochilco) (2024). Anuario de Estadísticas del Cobre y Otros Minerales 2005–2024.

[17] Servicio Nacional de Geología y Minería (SERNAGEOMIN) (2025). Anuario de la Minería de Chile 2024.

[18] Jeldres R.I., Forbes L., Cisternas L.A. (2016). Effect of seawater on sulfide ore flotation: A review. Miner. Process. Extr. Metall. Rev..

[19] Mu Y., Peng Y. (2019). The effect of saline water on copper activation of pyrite in chalcopyrite flotation. Miner. Eng..

[20] Millero F.J. (2013). Chemical Oceanography.

[21] Li Y., Li W., Xiao Q., He N., Ren Z., Lartey C., Gerson A. (2017). The influence of common monovalent and divalent chlorides on chalcopyrite flotation. Minerals.

[22] Cruz C., Botero Y.L., Jeldres R.I., Uribe L., Cisternas L.A. (2022). Current status of the effect of seawater ions on copper flotation: Difficulties, opportunities, and industrial experience. Miner. Process. Extr. Metall. Rev..

[23] Castro S. (2018). Physico-Chemical Factors in Flotation of Cu-Mo-Fe Ores with Seawater: A Critical Review. Physicochem. Probl. Miner. Process..

[24] Hwang M., Mu Y., Cao L., Peng Y. (2024). Identifying True Collectorless Flotation of Chalcopyrite in Calcium Chloride Solutions. Miner. Eng..

[25] Yang X., Bu X., Xie G., Chelgani S.S. (2021). A comparative study on the influence of mono-, di-, and trivalent cations on the chalcopyrite and pyrite flotation. J. Mater. Res. Technol..

[26] Suyantara G.P., Hirajima T., Miki H., Sasaki K. (2018). Floatability of Molybdenite and Chalcopyrite in Artificial Seawater. Miner. Eng..

[27] Uribe L., Gutierrez L., Laskowski J., Castro S. (2017). Role of calcium and magnesium cations in the interactions between kaolinite and chalcopyrite in seawater. Physicochem. Probl. Miner. Process..

[28] Hirajima T., Suyantara G.P., Ichikawa O., Elmahdy A.M., Miki H., Sasaki K. (2016). Effect of Mg^2+^ and Ca^2+^ as divalent seawater cations on the floatability of molybdenite and chalcopyrite. Miner. Eng..

[29] Li W., Li Y. (2019). Improved understanding of chalcopyrite flotation in seawater using sodium hexametaphosphate. Miner. Eng..

[30] Cui W., Chen J. (2021). Insight into mineral flotation fundamentals through the DFT method. Int. J. Min. Sci. Technol..

[31] Luo Y., Xia Y., Zhou H., Yin C., Yang H., Chen J., Ou L. (2022). Effect of calcium ions on surface properties of chalcopyrite and arsenopyrite and its response to flotation separation under low-alkalinity conditions. Appl. Surf. Sci..

[32] Li W., Li Y., Xie S., Duan W., Chen W. (2022). Roles and Influences of Kerosene on Chalcopyrite Flotation in MgCl_2_ Solution: EDLVO and DFT Approaches. Minerals.

[33] Hall S.R., Steward J.M. (1973). The crystal structure refinement of chalcopyrite, CuFeS_2_. Acta Crystallogr. Sect. B.

[34] Liu Y., Chen J., Li Y., Zhao C. (2023). A First-Principles Study on the Coadsorption of H_2_O and O_2_ on the Chalcopyrite (112)-M Surface. Int. J. Min. Sci. Technol..

[35] Laskowski J.S., Castro S., Ramos O. (2014). Effect of Seawater Main Components on Frothability in the Flotation of Cu–Mo Sulfide Ore. Physicochem. Probl. Miner. Process..

[36] Clark S.J., Segall M.D., Pickard C.J., Hasnip P.J., Probert M.I., Refson K., Payne M.C. (2005). First principles methods using CASTEP. Z. Kristallogr..

[37] Xie Y., Ban X., Yin W., Song N., Yao J. (2024). The application of KMnO_4_ in reverse flotation separation of chalcopyrite and talc and its selective depression mechanism. J. Environ. Chem. Eng..

[38] Ge W., Gao P., Liu J., Yuan S., Ren H., Zhu Y., Han Y., Li Y., Cheng Z. (2025). Experimental and theoretical studies on the effect of Fe^3+^ on the surface properties and floatability of chalcopyrite. Appl. Surf. Sci..

[39] Liu Z., Liu D., Liu Y., Xu L., Wen S. (2025). Selective depressing of chalcopyrite and molybdenite flotation by captopril: Mechanisms and insights. Appl. Surf. Sci..

